# An Early Neurological Indicator of Immune Effector Cell-Associated Neurotoxicity Syndrome

**DOI:** 10.7759/cureus.57298

**Published:** 2024-03-30

**Authors:** Yasufumi Yorichika, Shuichiro Neshige, Taro Edahiro, Shiro Aoki, Hirofumi Maruyama

**Affiliations:** 1 Department of Clinical Neuroscience and Therapeutics, Hiroshima University Graduate School of Biomedical and Health Sciences, Hiroshima, JPN; 2 Department of Hematology, Hiroshima University Graduate School of Biomedical and Health Sciences, Hiroshima, JPN

**Keywords:** diffuse large b-cell lymphoma (dlbcl), cytokine release syndrome (crs), mri, car-t cell therapy, immune effector cell-associated neurotoxicity syndrome (icans)

## Abstract

We herein report a 58-year-old female patient undergoing chimeric antigen receptor T-cell (CAR-T) therapy for refractory diffuse large B-cell lymphoma (DLBCL). Following the CAR-T infusion, the patient experienced Cytokine Release Syndrome (CRS), which was subsequently remitted. However, aphasia was observed five days post-infusion, and a loss of consciousness occurred on the sixth day. Brain MRI revealed a possibly high signal intensity in the mesial temporal region. The patient was diagnosed with immune effector cell-associated neurotoxicity syndrome (ICANS) secondary to CRS and received treatment with dexamethasone, which promptly improved her consciousness. As the diagnosis of ICANS was confirmed following the emergence of aphasia, vigilant cognitive monitoring of cognitive function is crucial in patients following CAR-T therapy.

## Introduction

Chimeric antigen receptor T-cell (CAR-T) therapy is a promising treatment for relapsed or refractory diffuse large B-cell lymphoma (DLBCL), showing higher response rates compared to conventional chemotherapy [1-3]. However, it can be associated with severe side effects, one of which is immune effector cell-associated neurotoxicity syndrome (ICANS), presenting symptoms ranging from nonspecific ones like headache and fever to potentially fatal outcomes such as impaired consciousness, seizures, and cerebral edema [4]. Mortality rates due to these severe complications can reach up to 3% [4,5]. Although the exact pathophysiology of ICANS is not fully understood, it is conceivable that ICANS is related to cytokine release syndrome (CRS) [6]. Therefore, early detection of this condition and therapeutic intervention are crucial. We herein report a case of ICANS following CRS to highlight the importance of early neurological symptoms for immediately identifying ICANS.

## Case presentation

**Figure 1:**
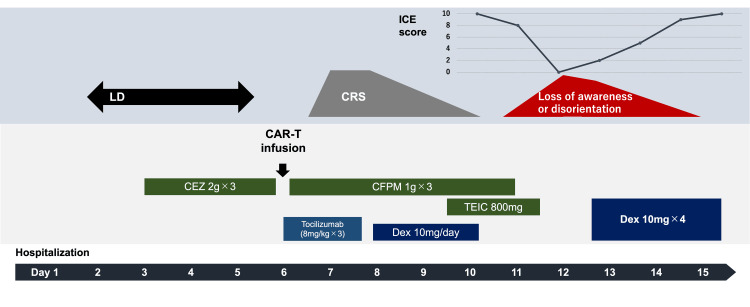
Clinical course of the patient The patient’s symptoms and treatment history are shown, along with the ICE score change. CAR-T, chimeric antigen receptor T-cell; CRS, cytokine release syndrome; CEZ, cefazolin; CFPM, cefepime; Dex, dexamethasone; ICE, immune effector cell-associated encephalopathy; TEIC, teicoplanin; LD, lympho-depletion chemotherapy

A 58-year-old woman with a history of DLBCL diagnosed seven years earlier, which was in complete remission after autologous peripheral blood stem cell transplantation, presented with relapsed disease marked by left submandibular lymph node swelling. As four cycles of R-GCD (rituximab, gemcitabine, carboplatin, dexamethasone) therapy showed limited effect, CAR-T therapy was considered, leading to her admission to the hematology department. Lympho-depletion chemotherapy began on the second day of hospitalization, with CAR-T cell infusion following on the sixth day. Subsequently, she developed a high-grade fever, leading to a diagnosis of CRS. Treatment included noradrenaline for circulatory support and tocilizumab (8 mg/kg, every eight hours) followed by dexamethasone (10 mg/day), which stabilized her condition (Figure 1).

On the 10th day post-admission, her consciousness was clear. However, on day 11, although her Glasgow Coma Scale (GCS) score was 15, her overall level of responsiveness was slightly reduced. More specifically, she became incapable of writing and was unable to perform calculations. Thus, the ICE (immune effector cell-associated encephalopathy) score was eight due to the inability to write and calculate. On day 12 of hospitalization, she was referred to a neurologist because of progressive loss of consciousness and a decrease in ICE score from eight to zero.

**Figure 2:**
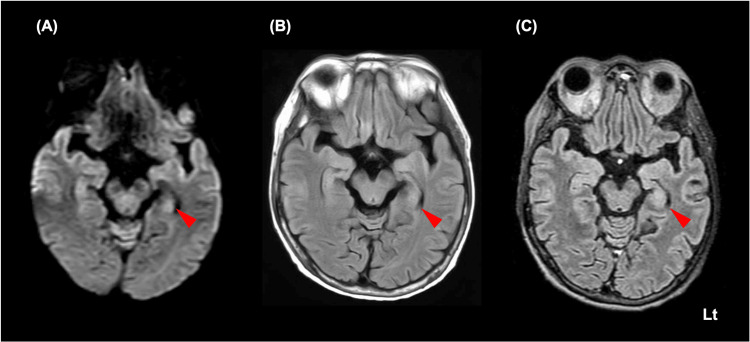
Brain magnetic resonance imaging Although prominent abnormal findings are not visible, there might be high intensity in the left mesial temporal region (red arrowhead) in the diffusion-weighted imaging (A), T2-weighted FLAIR imaging (B), and contrast-enhanced 3D FLAIR imaging (C). FLAIR, fluid-attenuated inversion recovery

On examination, her body temperature was 36.9°C, blood pressure was 108/69 mmHg, pulse rate was 93/min, and her GCS score was 11 (E3V3M5). Neurological examination revealed attention disorder and motor aphasia, along with bilateral upper limb myoclonus during posture. Signs of meningeal irritation were not observed. Blood tests showed unremarkable findings other than pancytopenia (white blood cell: 2.23×10^3^/μL, hemoglobin: 8.7 g/dL, platelet: 41×10^3^/μL). There were no obvious abnormalities that could explain her impaired consciousness. CSF examination was not performed due to low platelet counts. Brain MRI showed no findings suggestive of central nervous system involvement or stroke (Figure 2).

However, there might be high intensity in the left mesial temporal region. Therefore, based on the American Society for Transplantation and Cellular Therapy (ASTCT) criteria, a diagnosis of ICANS following CRS was established, necessitating dexamethasone 10 mg every six hours administration on day 13 of admission for grade 3 ICANS. After receiving dexamethasone, the patient regained consciousness, and the ICE score improved to 10 on day 15. The patient did not experience any ICANS flare-ups. Although her neurological symptoms dramatically resolved, she died on day 40 due to the progression of the DLBCL. As the family expressed their wish not to have an autopsy, no autopsy was carried out.

## Discussion

We report a case of ICANS characterized by impaired responsiveness with disorientation and aphasia secondary to CRS, which improved following dexamethasone treatment. We consider that early recognition of these neurological signs facilitated prompt inclusion in the differential diagnosis and early initiation of dexamethasone treatment. 

In this case, tisagenlecleucel was used as the product of CAR-T therapy, and the regimen is known to cause ICANS with a median of six days after the onset of CRS [2]. The patient showed impaired consciousness on day six of CRS onset, suggesting that the onset of ICANS occurred during the period of susceptibility for ICANS. Administration of intravenous dexamethasone 10 mg every six hours, as recommended in the guideline for ASTCT consensus grade 3 ICANS, resulted in a rapid improvement of symptoms [4,6]. Conversely, concerns have been raised that corticosteroids, including dexamethasone, may shorten progression-free survival, making their use controversial [7].

Four imaging patterns have been reported in ICANS: encephalitis, stroke, leptomeningeal disease, and posterior reversible encephalopathy syndrome [8]. However, MRI-negative cases occur in 13-69% of ICANS cases [5,6,8]. Patients with MRI abnormalities are assumed to be associated with poor outcomes, while those without MRI abnormalities are expected to recover from neurotoxicity [5]. Therefore, there may be a need for early diagnosis and therapeutic intervention before the disease presents structural changes on MRI. Nonetheless, the underlying mechanism and the specific localization within the brain responsible for aphasia, which emerges as the most prevalent symptom among patients with ICANS, remain unidentified. In the present case, the interpretation of MRI findings in the left mesial temporal region was controversial. Additionally, we did not confirm the post-treatment MRI findings. Should there have been a significant MRI abnormality, the early diagnosis and therapeutic intervention could potentially have impacted the patient's neurological outcome. 

High tumor burden and thrombocytopenia are recognized as risk factors for ICANS [6,9]. Rubin et al. reported that neurotoxicity was more likely to occur in the group with an early CRS onset (median day one) than in the group with a late CRS onset (median day four) [10]. They also reported that the group expressing severe neurotoxicity developed CRS earlier. In the present case, the onset of CRS occurred on the first day after infusion, suggesting that neurotoxicity was likely to develop and the patient was at high risk for severe disease.

## Conclusions

ICANS, a common adverse event of CAR-T therapy, remains poorly understood. Furthermore, as CAR-T therapy is expected to become more widely used for treating refractory and relapsed DLBCL, there is a critical need for enhanced risk stratification and management strategies. Therefore, the early detection of ICANS requires prompt recognition of chronological changes in neurological findings, such as aphasia.
